# Statement of retraction: Somatostatin attenuates intestinal epithelial barrier injury during acute intestinal ischemia–reperfusion through Tollip/Myeloiddifferentiationfactor 88/Nuclear factor kappa-B signaling

**DOI:** 10.1080/21655979.2025.2548109

**Published:** 2025-08-28

We, the Editor and Publisher of *Bioengineered*, have retracted the following article:

Tian, Y., Shu, R., Lei, Y., Xu, Y., Zhang, X., & Luo, H. (2022). Somatostatin attenuates intestinal epithelial barrier injury during acute intestinal ischemia–reperfusion through Tollip/Myeloiddifferentiationfactor 88/Nuclear factor kappa-B signaling. Bioengineered, 13(3), 5005–5020. https://doi.org/10.1080/21655979.2022.2038450

Following publication, the authors contacted the publisher to notify the journal that the study had failed its animal ethics approval review, and that there were additional concerns raised regarding the data. When contacted for further information, the authors were unable to address the concerns raised.

In February 2025, concerns were raised by a third party regarding a mismatch between the study described and the ethics approval statement in the article.

As verifying the validity of published work is core to the integrity of the scholarly record, we are therefore retracting the article. The corresponding author listed in this publication has been informed.

We have been informed in our decision-making by our editorial policies and the COPE guidelines. The retracted articles will remain online to maintain the scholarly record, but they will be digitally watermarked on each page as ‘Retracted’.

